# The Use of Fluorescence Spectrometry Combined with Statistical Tools to Determine the Botanical Origin of Honeys

**DOI:** 10.3390/foods13203303

**Published:** 2024-10-18

**Authors:** Aleksandra Wilczyńska, Natalia Żak

**Affiliations:** Department of Quality Management, Gdynia Maritime University, ul. Morska 81-87, 81-225 Gdynia, Poland; n.zak@wznj.umg.edu.pl

**Keywords:** authenticity of honey, fluorescence spectra (EEM), method of identification, varieties and origin of honey

## Abstract

At a time when the botanical origin of honey is being increasingly falsified, there is a need to find a quick, cheap and simple method of identifying its origin. Therefore, the aim of our work was to show that fluorescence spectrometry, together with statistical analysis, can be such a method. In total, 108 representative samples with 10 different botanic origins (9 unifloral and 1 multifloral), obtained in 2020–2022 from local apiaries, were analyzed. The fluorescence spectra of those samples were determined using a F-7000 Hitachi fluorescence spectrophotometer, Tokyo, Japan. It is shown that each honey variety produces a unique emission spectrum, which allows for the determination of its botanical origin. Taking into account the difficulties in analyzing these spectra, it was found that the most information regarding botanical differences and their identification is provided by synchronous cross-sections of these spectra obtained at Δλ = 100 nm. In addition, this analysis was supported by discriminant and canonical analysis, which allowed for the creation of mathematical models, allowing for the correct classification of each type of honey (except dandelion) with an accuracy of over 80%. The application of the method is universal (in accordance with the methodology described in this paper), but its use requires the creation of fluorescence spectral matrices (EEG) characteristic of a given geographical and botanical origin.

## 1. Introduction

According to its definition, honey is a product produced by honeybees, *Apis mellifera*, from nectar or honeydew and occupies a special place in the human diet [1]. Its role in human nutrition is due to its rich taste and nutritional effects on the human body. The health-promoting properties of honey are closely related to its chemical composition, which depends mainly on the botanical origin of the honey (honey variety) [2,3]. Honey comes in many different varieties, depending on the source of the nectar honey (nectar and honeydew honey), the botanical origin and the geographical location [3]. The classification of nectar honey into a given variety depends on the plant pollen that is dominant. For example, in Central Europe, the most common varieties of honey are rapeseed, lime, dandelion, goldenrod, buckwheat and honeydew (from honeydew from deciduous and coniferous trees), as well as less common ones, such as phacelia, raspberry and clover. In other geographical zones, eucalyptus, chestnut, rose, lavender, orange and other honeys characteristic of the vegetation occurring in a given geographical zone are found. Honey in which no pollen predominates is classified as multifloral. Each of these honeys, depending on the variety, production method and origin, will have different properties.

The specific taste and health-promoting properties of honey contribute to its high price. The desire to increase profits encourages producers or traders to adulterate honey. Adulteration is carried out, for example, by mixing honeys of different varieties, mislabeling them, adding sugar syrups, repeatedly heating honeys, and other processes used intentionally by producers [4]. However, recently, the most frequent fraud has been the mislabeling of the botanical origin of honeys [5]. In order to verify the quality of honeys and confirm their authenticity, a number of research methods are used. Mandatory methods for assessing the quality of honeys are usually described in legal acts. In Poland, this is the Regulation of the Minister of Agriculture and Rural Development, from 29 May 2015, amending the regulation on detailed requirements for the commercial quality of honey (Journal of Laws 2015, item 850). The only method recommended in legal acts to determine the botanical origin of honeys is pollen analysis. However, both in Poland and around the world, other, non-standard methods for assessing honey quality parameters are used, which can be used to determine not only the elementary chemical composition of individual honeys, but also the biological activity of honeys and their components [5,6,7,8,9,10,11,12]. As a result of the analysis of the literature on the assessment of the quality and authenticity of honeys, it was found that it is impossible to indicate repeatable, characteristic parameters, the value of which would allow for the unambiguous determination of the affiliation of a given sample of a specific honey variety. Various methods that have been used so far require not only financial outlays and time, but also confirmation of the accuracy of their result with alternative measurements. In many countries, compounds characteristic of a specific honey variety are sought, or the chemical profiles of a specific class of natural products are created (so-called “fingerprints” of individual honey varieties), but the results of these works are still unsatisfactory [4,5,12,13,14,15]. It can therefore be assumed that taking action to develop a reliable, fast and cheap method of assessing the quality and authenticity of honey will also allow for the detection and prevention of adulterated or lower-quality honey being introduced into circulation.

The use of excitation–emission matrices (EEMs) is one of two commonly used methods for measuring fluorescence spectra in the field of food safety and quality [16,17].

Although fluorescence spectroscopy techniques are widely used in the field of food quality and safety, they still have some limitations [18]. For example, Ruof et al. have been conducting research on the use of fluorescence-based methods for assessing the quality and identification of honey varieties for many years [19,20,21].

This study focused on refining the method and measurement conditions, as well as statistical tools for data analysis. The assumed effect of this study is a methodology, the application of which is to be a universal tool used on a wider scale. Creating fluorescence spectral matrices for honeys of various botanical origins will streamline the identification process, reduce the measurement time, and above all reduce the amount of reagents used and the costs incurred. Additionally, these tests are non-destructive with a high level of precision, sensitivity and repeatability of measurement performance. The preparation of the sample for testing does not require major expenditures. Therefore, the methodology can also be implemented to assess the quality of other parameters, such as the degree of filtration, honey aging, its overheating or its storage in improper conditions [22,23,24].

Therefore, the aim of our work was to demonstrate that fluorescence spectrometry can be such a method. Fluorescence is one of the physical techniques that is widely used for food authentication. Fluorescence spectra and images may both be considered unique sample fingerprints. This analytical technique is non-destructive, rapid and sensitive, especially when combined with multivariate analysis tools such as principal component analysis, parallel factor analysis and factorial discriminant analysis [25,26]. Numerous studies indicate that it can be used to mark the authenticity of olive oils, other edible oils, wines, spices, fish products and other foods [27,28,29,30,31,32]. In recent years, there have been many reports about using spectroscopic techniques to analyze honey as an alternative to time-consuming conventional methods. Fluorescence spectroscopy was used, among others, to determine the quality and authenticity of honey, but also to detect various contaminants or adulterants [33,34,35,36,37,38,39]. However, many of these reports show that determining the botanical origin of honey based only on fluorescence spectra is impossible. Therefore, we decided to demonstrate that such identification is possible both on the basis of 3D and synchronous spectra, but above all on the basis of differences in the fluorescence intensity of honeys of different varieties in the entire emission band, at a specified Δλ = 100 nm.

## 2. Materials and Methods

In total, 108 honey samples of 10 different botanical origins (multifloral: 14, honeydew: 12, acacia: 8, honeydew coniferous: 8, nectar honeydew: 8, rape: 13, phacelia: 9, lime: 14, buckwheat: 13, dandelion: 10, heather: 9) were analyzed to evaluate their fluorescence spectra. The honeys, provided by a local beekeepers’ association from the Pomeranian province, were harvested in 2020–2022. Until the analyses were performed, the tested samples were stored in tightly closed individual packages—250 mL glass jars with a “Twist off” closure, at a temperature of 16 to 20 °C.

Fluorescence spectra were determined by a method patented by Gębala and Przybyłowski [40,41].

Excitation–emission matrices, EEMs, were acquired by a series of emission scans measured over a range of excitation wavelengths to create a fluorescence contour map.

The tests were performed using a set consisting of the following:

1. A Hitachi F-7000 fluorescence spectrophotometer, Tokyo, Japan;
2. A specially constructed adapter for measuring fluorescence intensity measured from the sample surface to change its traditional measurement range.

**Figure 1:**
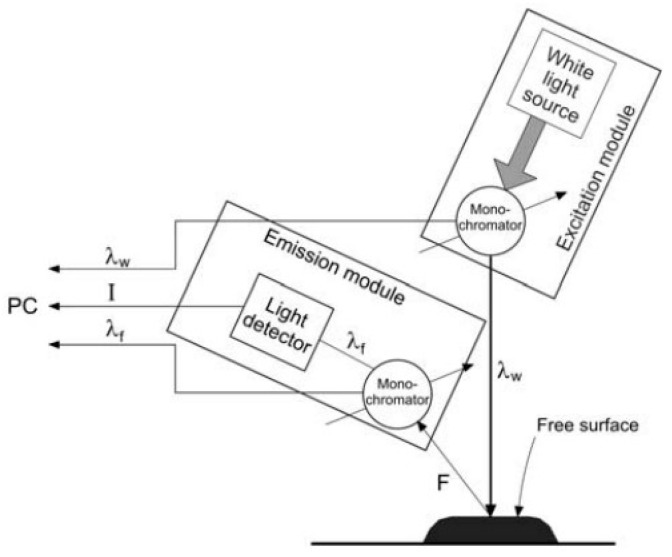
Scheme of surface fluorescence measurement. Source: (adapted from: [40,41]).

Figure 1 shows how the energy of the light beam reappears and re-emits at lower quanta after being reflected from the surface of the tested sample.

The sample surface is exposed to excitation radiation and the reflective geometry allows us to eliminate the effects of the internal filter associated with the high absorbance of the sample—the weakening of fluorescence intensity is due to the excitation absorbance and the emitted radial radiation.

Dimensional fluorescence spectra were measured at room temperature and in daylight. Honey samples were condensed (temp. 40 °C) and pipetted into 0.5 mL quartz cuvettes before measurement. Fluorescence spectra were obtained by recording emission spectra (from 220 to 560 nm with 10 nm steps) corresponding to excitation wavelengths in the range from 200 to 450 nm (with 5 nm steps) and automatically normalized to the excitation intensity of the instrument. The voltage used to determine the sensitivity of the excitation and emission measurements was equal to 600 V. The difference between the wavelength of fluorescent light (γF) and the wavelength of excitation light (γw) was preferentially 100 nm [40,41]. In order to reduce scattering effects and compare the honey samples studied, the fluorescence spectra were normalized by reducing the area under each spectrum to the value of 1 [22].

All analyses were performed in triplicate. The final results are presented as a set of numerical data in the form of contour maps (excitation emission (EEM)) and the synchronous cross-sections of these spectra were obtained at Δλ = 100 nm for honey samples.

### Statistical Analysis

To determine the botanical origin on the shape of the spectra, a discriminant analysis was performed. The analysis was performed using the Statistica 13.3 multivariate discriminant analysis package.

This article uses discriminant analysis, which is a set of statistical methods for multidimensional data analysis. Its main goal is to decide which independent variables affect the classification of the described dependent variables. The classical discriminant analysis used in this study allows us to build a forecast model of group membership. This model is created on the basis of a discriminant function that provides the best distinction between groups. The sample of observations used to generate the function is known. It is an extremely effective tool used for classification issues and data exploration. Its advantage is a high level of effectiveness for homogeneous data, while its disadvantage is a lack of effectiveness on non-homogeneous data.

This analysis was aimed at confirming the hypothesis that the identification of honey varieties is possible based on fluorescence spectra. It was therefore checked whether the compared varieties differed in terms of the value of the mean fluorescence intensity within the entire emission band or in part of it at Δλ 60 nm, 80 nm and 100 nm. However, attempts to perform calculations at Δλ 60 nm and 80 nm did not produce results that provided statistically significant information. Then, appropriate canonical discriminant functions were constructed. Statistical hypotheses were verified at a significance level of *p* = 0.05.

## 3. Results

### 3.1. Analysis of Spectra

The emission spectra (excitation: 200–450 nm; emission: 260–560 nm) considered in this investigation allowed for the study of the fluorescence of the honey samples. The complete fluorescence spectra of different botanical origins of honey are characterized by a spectral region of high emission intensity, originating from fluorophores such as phenolic compounds and aromatic amino acids [26].

**Figure 2:**
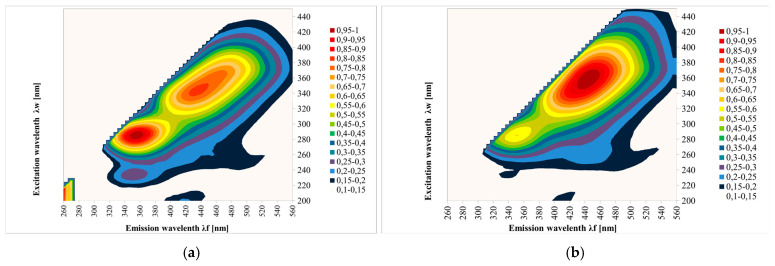

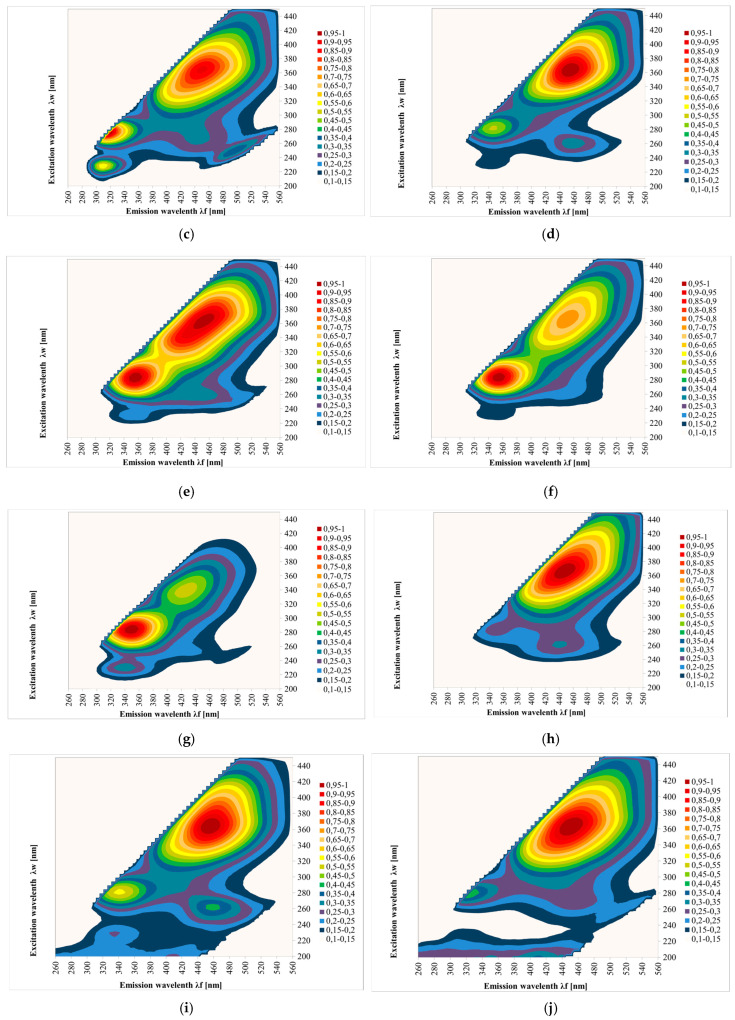
Excitation emission (EEM) spectra of different botanical origins of honey; (**a**)—acacia, (**b**)—phacelia, (**c**)—buckwheat, (**d**)—linden, (**e**)—dandelion, (**f**)—nectar–honeydew, (**g**)—rapeseed, (**h**)—honeydew–coniferous, (**i**)—multifloral, (**j**)—heather. Source: own research.

Figure 2 shows the EEM spectra measured for the tested honeys. The results show contour maps for 108 samples of different honey types: multifloral, acacia, honeydew coniferous, nectar honeydew, rape, phacelia, lime, buckwheat, dandelion and heather. The fluorescence measurement of the sample surface was performed three times.

### 3.2. Discrimination of Honeys by Mathematical Method

In the next part, a discriminant analysis was performed to confirm the hypothesis that the identification of honey varieties is possible based on fluorescence spectra. It was therefore checked whether the compared varieties differ in terms of the value of the average fluorescence intensity within the entire emission band or in part of it at Δλ 100 nm. Then, appropriate canonical discriminant functions were constructed.

Based on the analysis of the constructed classification matrices, that the following was found:

- The selection of any model covering only a part of the emission band does not allow us to obtain a 100% correct classification of all honey samples;
- After introducing all of the measurement points of the emission band into the model, a 100% qualification of varietal honeys was obtained at Δλ 100 nm within the entire emission band (Table 1).

The parameters of the measurements of the excitation waves of honeys for Δλ = 100 nm of different botanical origins were different. This allowed for the discrimination of the studied population of honeys by variety, which was statistically confirmed by the Wilks lambda test (F = 3.91, *p* = 0.0000001), assuming a test probability value of *p* ≤ 0.05. This means that based on the measurement of the fluorescence intensity of honeys, their botanical identification was achieved in the case of 80.0% of the tested samples (the identification of dandelion honey may be difficult because it is located in the center of the coordinate system and close to three other varieties).

The main stage of the discriminant analysis was performed using the standard method. The canonical analysis performed next allowed for the calculation of raw coefficient values (F_1_ and F_2_) of the discriminant function for the first two roots and the construction of canonical discriminant functions.

The canonical analysis included also the calculation of the coefficients of canonical variables (D_1_ and D_2_) and their average values (Table 2).

**Figure 3:**
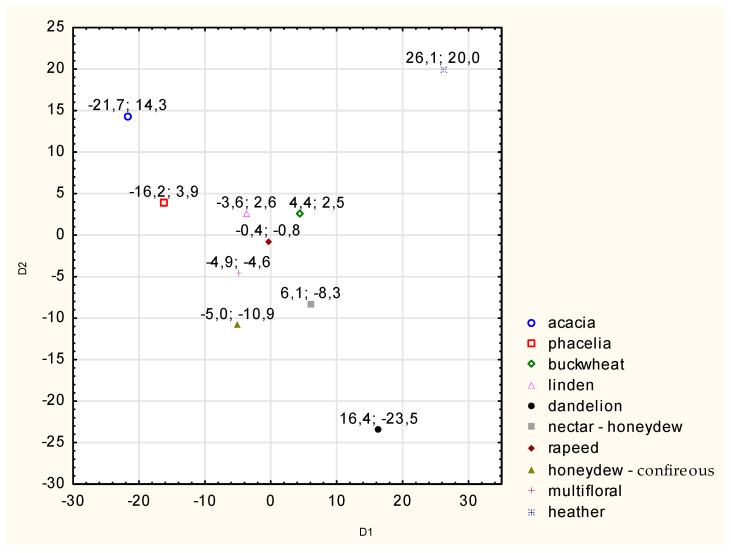
Analysis of the fluorescence intensity of honeys of different varieties in the entire emission band at Δλ = 100 nm—canonical variate averages. Source: own research.

The canonical analysis performed allowed for a graphical presentation of the results of the calculations of the canonical mean values (Figure 3). It illustrates the position of individual honey varieties in a two-coordinate system (canonical variables).

Table 3 shows the outline of the spreadsheet file that can be used to calculate the values of two canonical variates—D_1_ and D_2_.

Using the above model, it is possible to identify all ten honey varieties using fluorescence spectra for Δ = 100 nm. To do this, you should do the following:

- Calculate the D_1_ and D_2_ values according to the scheme presented in Table 3;
- Compare their values with the values given in Table 2, which contains the values of the mean canonical variables.

**Figure 4:**
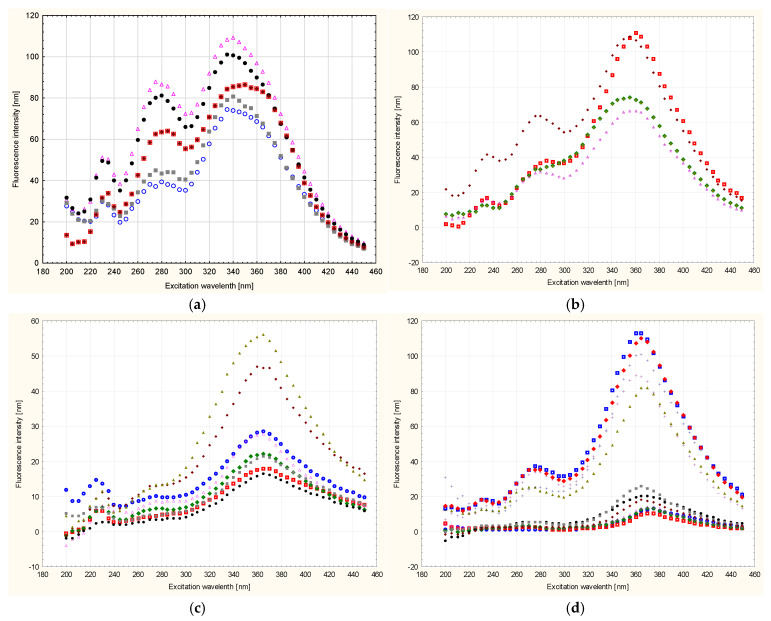

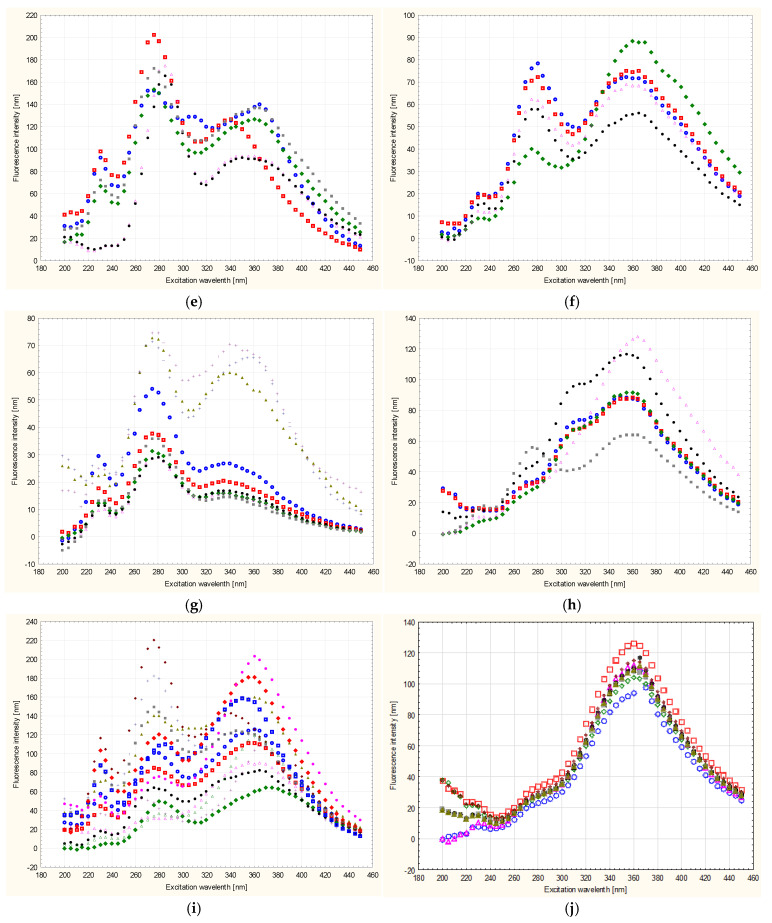
Synchronous fluorescence spectrum of honeys of different botanical origin at Δλ = 100 nm with each sample marked in the species group; (**a**)—acacia, (**b**)—phacelia, (**c**)—buckwheat, (**d**)—linden, (**e**)—dandelion, (**f**)—nectar–honeydew, (**g**)—rapeseed, (**h**)—honeydew–coniferous, (**i**)—multiflora, (**j**)—heather. Different colors shown in the figure indicate separate samples. Source: own research.

**Figure 5:**
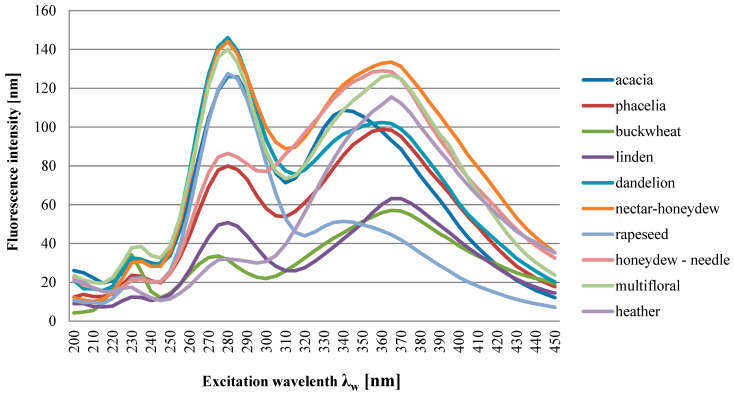
Synchronous fluorescence spectrum for varietal honeys at Δλ = 100 nm (mean value). Source: own research.

Based on the analysis carried out, taking into account the difficult identification and analysis of the spectra, it was found that the greatest essence of information concerning botanical differences and their identification is contained in the synchronous sections of these spectra obtained at Δλ = 100 nm, which are shown in Figure 4 and Figure 5. It can also be seen that the spectra of all tested honeys in a given variety group were characterized by the presence of emission bands of different intensities, but the same locations of the excitation wavelength Δλw for the maximum level of fluorescence intensity. Figure 4 shows the tested honeys, taking into account each of the tested samples in the variety group. Each sample was marked with a different marker in order to show the deviations of the fluorescence measurements in the variety group.

The spectra of all the honeys tested, depending on their botanical origin, were characterized by the presence of individual emission bands of different intensities. Additionally, the bands were distinguished by different locations of the excitation wavelength Δλw for the maximum level of fluorescence intensity at Δλ = 100 nm (Figure 5). It can be stated that in eight varieties, there were three excitations at different excitation wavelengths Δλw, which is also presented in more detail in Table 4. In the heather and honeydew–conifer varieties, there are two excitations at different excitation wavelengths Δλw, 235 nm and 360 nm for honeydew–conifer honeys and 370 nm for heather honeys. All the honey varieties tested had a visible tendency to increase the intensity of the shortwave emission excitation band at the level of 235 nm and 240 nm (phacelia). Another increase in intensity in the intermediate-range bands was found in eight varieties:

- Emission excitation: 280 nm (honey: acacia, buckwheat, lime, rapeseed, multifloral);
- Emission excitation: 285 nm (honey: phacelia, dandelion, nectar–honeydew).

The next increase in intensity in the bands in the long-wave spectral range (excitation of emission from 340 nm to 370 nm occurred in all ten varieties tested).

In our analysis, the synchronous spectrum of buckwheat and lime honey (Δλ = 100 nm) was characterized by a visible tendency to demonstrate three emission excitations, 235 nm, 280 nm and 370 nm, with different fluorescence emission intensities.

## 4. Discussion

It can be argued that the results of the use of fluorescence spectrometry in the form of total fluorescence spectra depend on the botanical origin of the honey, and this was confirmed by the studies presented by Ruoff et al. [19,20,21], Gębala [40], Gębala and Przybyłowski [41], Dramićanin et al. [39] and our former studies [22]. These authors also indicated small differences in fluorescence intensity in the tested group of varietal honeys. Gębala and Przybyłowski grouped the spectral features of Polish botanical honeys, taking into account the levels of excitation fluorescence intensity as indicators characteristic of varietal honeys found in Poland [40]. Pari et al. grouped samples of honeys of Italian origin into subsections of honeys of different botanical origins. The grouping was based on similar spectral features. The authors also point to the main components, the so-called fluorophores, which are responsible for the fluorescence emission of the honeys studied [33]. In 2014, Nikolova’s team attempted to compare the spectral characteristics of honeys adulterated by the addition of sweeteners. The results obtained indicated that the study of fluorescence spectra opens up the possibility of distinguishing honey samples with added artificial sweeteners from natural ones [42]. In our previous studies, we attempted to demonstrate that fluorescence spectra can be used to indicate the degree of overheating of honey [23]. We also obtained confirmation that honey filtration does not affect the classification of honey spectra of various botanical origins [22]. According to the study conducted by Dramićanin et al. [39], the differences in the fluorescence of natural and adulterated honey samples are extremely significant in five spectral regions due to the differences in the concentrations and local environments of aromatic amino acids, phenolic compounds, furosine and hydroxymethylfurfural. This was also confirmed by statistical tests and PCA. According to these authors, by quantifying the fluorescence responses and subjecting them to a statistical classification technique, for example, LDA, it is possible to detect adulterated honey with 100% accuracy. Such accuracy suggests that fluorescence excitation emission spectroscopy may be a promising method for low-level honey adulteration, which will be the subject of our future work.

## 5. Conclusions

In our study, 108 pure honey samples from 10 different botanical origins have been analyzed using fluorescence emission. We have shown that each honey variety is characterized by a unique total spectrum, enabling the classification of their botanical origin. However, taking into account the difficulties in analyzing these spectra, it was found that the most information regarding botanical differences and their identification is provided by synchronous cross-sections of these spectra obtained at Δλ = 100 nm. Additionally, discriminant analysis showed that it was possible to identify most of the tested honey varieties; the identification of dandelion honey will be problematic.

## Figures and Tables

**Table 1:** Honey variety classification matrix at Δλ 100 nm in the entire emission band.

Honey	% Correct Classification	Acacia	Phacelia	Buckwheat	Linden	Dandelion	Nectar–Honeydew	Rapeseed	Honeydew–Confireous	Multifloral	Heather
acacia	100	6	0	0	0	0	0	0	0	0	0
phacelia	100	0	6	0	0	0	0	0	0	0	0
buckwheat	100	0	0	8	0	0	0	0	0	0	0
linden	100	0	0	0	12	0	0	0	0	0	0
dandelion	100	0	0	0	0	6	0	0	0	0	0
Nectar–honeydew	100	0	0	0	0	0	5	0	0	0	0
rapeseed	100	0	0	0	0	0	0	10	0	0	0
Honeydew–needle	100	0	0	0	0	0	0	0	6	0	0
multifloral	100	0	0	0	0	0	0	0	0	14	0
heather	100	0	0	0	0	0	0	0	0	0	8

Source: own research.

**Table 2:** Analysis of fluorescence intensity in the entire emission band at Δλ = 100 nm—averages of canonical variables.

Canonical Variables	Honey									
	Acacia	Phacelia	Buckwheat	Linden	Dandelion	Nectar–Honeydew	Rapeseed	Honeydew–Confireous	Multifloral	Heather
	Averages of Canonical Variates									
D_1_	−21.71	−16.19	4.434	−3.644	16.38	6.091	−0.367	−4.99	−4.901	26.144
D_2_	14.3	3.88	2.54	2.564	−23.47	−8.336	−0.778	−10.86	−4.607	19.975

Source: own research.

**Table 3:** Analysis of the fluorescence intensity of honeys of different varieties in the entire emission band at Δλ = 100 nm—scheme for calculating the value D_1_ and D_2_.

Emission Band	Fluorescence Intensity	F_1_ (Discriminant Function Coefficients)	Square 1× Fluorescence Intensity	F_2_ (Discriminant Function Coefficients)	Square 2× Fluorescence Intensity
Free term	-	−3.664	−3.664	2.677	2.677
200.000		−0.502		0.079	
205.000		−0.717		−0.577	
210.000		1.398		0.927	
215.000		−1.146		−1.452	
220.000		1.239		1.299	
225.000		1.425		0.840	
230.000		0.824		−0.461	
235.000		−2.946		−2.929	
240.000		−0.666		4.322	
245.000		3.522		−1.546	
250.000		−0.464		0.131	
255.000		−1.920		−0.054	
260.000		−0.696		−2.729	
265.000		−2.201		2.347	
270.000		1.950		1.718	
275.000		0.763		−3.293	
280.000		2.151		1.787	
285.000		−1.074		0.707	
290.000		0.056		−0.231	
295.000		−3.589		−2.249	
300.000		1.211		−0.335	
305.000		2.889		3.227	
310.000		−1.094		−1.092	
315.000		−1.987		−2.966	
320.000		−0.798		0.206	
325.000		4.240		1.035	
330.000		−0.729		4.727	
335.000		−1.849		−3.363	
340.000		1.717		−1.704	
345.000		1.834		4.251	
350.000		−3.414		−2.002	
355.000		−4.152		−2.765	
360.000		4.684		1.588	
365.000		−0.456		0.161	
370.000		6.187		3.346	
375.000		−4.361		−5.826	
380.000		−4.968		2.121	
385.000		5.438		3.984	
390.000		−2.449		−3.884	
395.000		0.741		3.593	
400.000		−5.072		−4.049	
405.000		4.449		−0.149	
410.000		2.754		2.695	
415.000		−6.915		−0.923	
420.000		2.698		−1.150	
425.000		3.570		−0.825	
430.000		−1.298		2.171	
435.000		4.690		−4.005	
440.000		−1.523		0.744	
445.000		−8.798		−0.297	
450.000		5.980		3.072	
			TOTAL = D_1_		TOTAL = D_2_

Source: own research.

**Table 4:** Comparison of excitation wavelengths Δλw for the maximum fluorescence intensity of honeys of different botanical origin at Δλ = 100 nm—average.

Botanical Origin of Honey	Excitation Wavelength Δλw for Maximum Fluorescence Intensity of Varietal Honeys [nm]		
	I Excitation	II Excitation	III Excitation
acacia	235	280	340
phacelia	240	285	360
buckwheat	235	280	370
linden	235	280	370
dandelion	235	285	365
nectar–honeydew	235	285	360
rapeseed	235	280	340
honeydew–needle	235	absence	360
multifloral	235	280	365
heather	235	absence	370

Source: own research.

## Data Availability

The original contributions presented in the study are included in the article, further inquiries can be directed to the corresponding author.
